# Association between fusion visual function deficits and myopia in school-aged children: an analysis based on competitive binocular vision screening

**DOI:** 10.1186/s12886-026-04660-9

**Published:** 2026-02-09

**Authors:** Chen Chen, Xiaohan Zhang, Yingqing Yu

**Affiliations:** Eye Institute, Affiliated Children’s Hospital of Jiangnan University, Wuxi, Jiangsu Province 214023 People’s Republic of China

**Keywords:** Pediatric myopia, Fusion vision, Binocular vision disorders, Complex visual environment, Dynamic visual screening

## Abstract

**Objective:**

Even after refractive correction, myopic children remain at a significantly higher risk of visual function deficits compared to non-myopic children. Competitive binocular visual function testing is a screening method designed to simulate complex visual environments, specifically developed for the large-scale and rapid assessment of children’s visual performance under challenging conditions. This study aims to investigate whether visual function deficits observed in simulated complex environments are associated with myopia, thereby informing potential strategies for myopia prevention and control.

**Methods:**

A cross-sectional study was conducted involving 1,430 school-aged children from grades 1 to 6 at an elementary school in Wuxi in 2022. Competitive binocular visual functions (simultaneous perception, fusion, and stereopsis) were assessed and compared between myopic and non-myopic children.

**Results:**

The rate of fusion function deficits in the myopic group (35.80%, 256/715) was significantly higher than that in the non-myopic group (26.57%, 190/715). Myopic children had a 1.54 times higher risk of fusion deficits compared to non-myopic children (OR=1.54, 95% CI: 1.22–1.94). A weak positive correlation was observed between the degree of myopia and fusion deficits (r=0.087, P<0.01). Age stratification revealed that the fusion deficit rate in the lower-grade group (7–9 years old) (50.62%, 365/721) was significantly higher than that in the higher-grade group (10–12 years old) (11.42%, 81/709). Younger children had an 8.05 times higher risk of fusion deficits compared to older children (OR=8.05, 95% CI: 6.18–10.49). Gender differences showed that the fusion deficit rate in males (46.89%, 332/708) was significantly higher than that in females (15.79%, 114/722), with males having a 4.63 times higher risk than females (OR=4.63, 95% CI:3.64–5.89). No significant differences were observed in simultaneous perception or stereopsis between the myopic and non-myopic groups (P > 0.05).

**Conclusion:**

Myopic children are more likely to exhibit deficits in competitive binocular fusion function. These findings indicate that early childhood may represent a potential critical period for intervention in fusion function development.

**Supplementary Information:**

The online version contains supplementary material available at 10.1186/s12886-026-04660-9.

In recent decades, myopia has become a global public health issue, particularly prominent in East Asian populations, including China. The onset of myopia is a complex process involving both genetic predisposition and environmental influences. While extensive screen time and limited outdoor activities are recognized as significant behavioral risk factors, the exact pathogenesis remains incompletely understood. A growing body of evidence suggests that deficits in binocular visual functions, such as accommodative lag and convergence insufficiency, may contribute to the development and progression of myopia [1, 2]. Contemporary research, including recent reports from the International Myopia Institute (IMI), has further emphasized the role of dynamic visual processing and binocular coordination in myopia pathogenesis [3].

Binocular visual functions include simultaneous perception, fusion, stereopsis, and eye movement coordination. The development of these functions is crucial for children’s learning and daily activities. However, traditional research on myopia has primarily focused on changes in refractive parameters, with limited investigation into how higher-order visual functions, particularly under demanding conditions, influence myopia. Recent studies have begun to explore the link between accommodation/convergence and myopia progression. For instance, a longitudinal study found that an increased accommodative convergence/accommodation (AC/A) ratio served as an early sign of myopia and was associated with greater accommodative lag [3]. Another study indicated that myopic children exhibit high levels of accommodative adaptation, accompanied by accommodative lag and a high AC/A ratio, which deviates from the pattern predicted by current models of accommodation and convergence interaction [4, 5]. Despite these insights, few large-scale, school-based studies have specifically examined rivalry-state or competitive binocular visual function—the ability to maintain binocular coordination under conditions of visual conflict or distraction—in myopic children, a gap this study seeks to address.

Visual environments in modern life are increasingly complex and dynamic, characterized by frequent shifts between near and far tasks, high visual clutter, and digital device use, which may impose significant demands on the developing visual system. To assess visual performance under such realistic, challenging conditions. Our research team has developed an innovative screening protocol simulating complex visual environments, specifically designed for large-scale and rapid assessment of students’ visual performance under challenging conditions. We integrated standard vernier acuity and three-level binocular function tests (simultaneous perception, fusion, and stereopsis) with competitive binocular tasks—where conflicting stimuli are presented to each eye—to create a comprehensive visual function screening battery. This approach evaluates the risk of visual distortion and binocular imbalance in suboptimal viewing conditions, mirroring real-world challenges such as those encountered during prolonged digital device use. This approach evaluates the risk of visual distortion and binocular imbalance in suboptimal viewing conditions, mirroring real-world challenges such as those encountered during prolonged digital device use. The protocol comprises four key tests: competitive vernier acuity, competitive simultaneous perception, competitive fusion, and competitive stereopsis (see Fig. 1, which illustrates the test paradigm). Together, these tasks assess a student’s resilience and adaptability in dynamic visual settings.

This study involved 1,430 students from an elementary school in Wuxi, conducting an in-depth analysis of the differences in three core visual functions (simultaneous perception, fusion, and stereopsis) between myopic and non-myopic students. The primary aim is to reveal whether visual function deficits observed under simulated complex environmental conditions are associated with myopia, thereby providing evidence to inform future myopia prevention and control strategies.

## Materials and methods

Study Participants: This study selected students in grades 1 to 6 from a standard six-year elementary school in Wuxi in June 2022. A total of 1,430 students who completed all examinations were included. Inclusion criteria: (1) Age 7–12 years, able to cooperate in completing refractive error measurements and visual function tests; (2) Guardians provided signed informed consent; (3) All parents or legal guardians of any participant under the age of 16 consent to participate in this study. Exclusion criteria: (1) Incomplete refractive error or visual function examination data; (2) Presence of organic eye diseases such as strabismus, amblyopia, congenital cataracts, or retinopathy; (3) Cognitive impairment or inability to understand test instructions. This study adhered to the principles of the Declaration of Helsinki and was approved by the Ethics Committee of Wuxi Children’s Hospital (Ethics Approval No.: WXCH-2025-10-170). Guardians provided signed informed consent.

## General data and refractive measurements

### Refractive error measurement

Three certified ophthalmologists performed binocular refractive error measurements using a Canon R-F10 autorefractor without cycloplegia, a pragmatic choice for large-scale school-based screening. Each eye was measured three consecutive times. Valid data with a coefficient of variation (CV) < 5% were selected, and the average value was taken as the final refractive error for that eye. During measurements, participants were instructed to sit upright, focus on the built-in target of the instrument, and avoid blinking or head movement.

### Spherical equivalent (SE) calculation and grouping criteria

SE Calculation Method: The average value of both eyes was used as the indicator of individual refractive status, calculated as: SE = spherical power + 1/2 × cylindrical power.

Refractive Status Classification: Based on the myopia definition standard published by the International Myopia Institute (IMI) in 2021 [5], and considering children’s refractive development characteristics, participants were divided into the following groups: Non-myopia group: SE ≥ -0.50 D (including emmetropia, hyperopia, and mild hyperopic reserve); Myopia group: SE < -0.50 D, further subdivided into: Low myopia group: -0.50 D > SE ≥ -3.00 D; Moderate myopia group: -3.00 D > SE ≥ -6.00 D; High myopia group: SE < -6.00 D.

### Correction method

All participants wore optimal correction devices for best-corrected visual acuity (BCVA) during testing: Students with existing glasses: Wore their daily corrective glasses (lens power consistent with refractive results within the last 3 months, no significant wear); Students without glasses: Used trial frames (HOYA trial lens set, Japan) and underwent subjective refraction to achieve optimal correction, ensuring distant visual acuity (5 m) ≥ 0.8.

## Stimuli and procedure rivalry-state visual task test

The competitive-state visual function screening protocol used in this study is based on a visual assessment paradigm previously developed and preliminarily validated by our team [6]. The core task logic of the competitive fusion and stereopsis tests in this protocol is functionally isomorphic to clinically common tests such as the Worth four-dot test (assessing simultaneous perception and fusion) and random-dot stereograms (assessing stereopsis). It aims to simulate real-world visual interference by introducing dynamic competitive stimuli. The test was conducted in a quiet, private room with natural and constant lighting. A 32-inch adjustable-height monitor (resolution 1920 × 1080 pixels) was used to present stimulus images at a viewing distance of 80 centimeters. Participants wore corrective glasses (if needed) overlaid with red-blue glasses (red lens on the left, blue lens on the right) and stood upright for the test, with their eyes level with the center of the screen. (Note: This series of stimulus images can also be used on polarized 3D displays).

**Fig. 1:**
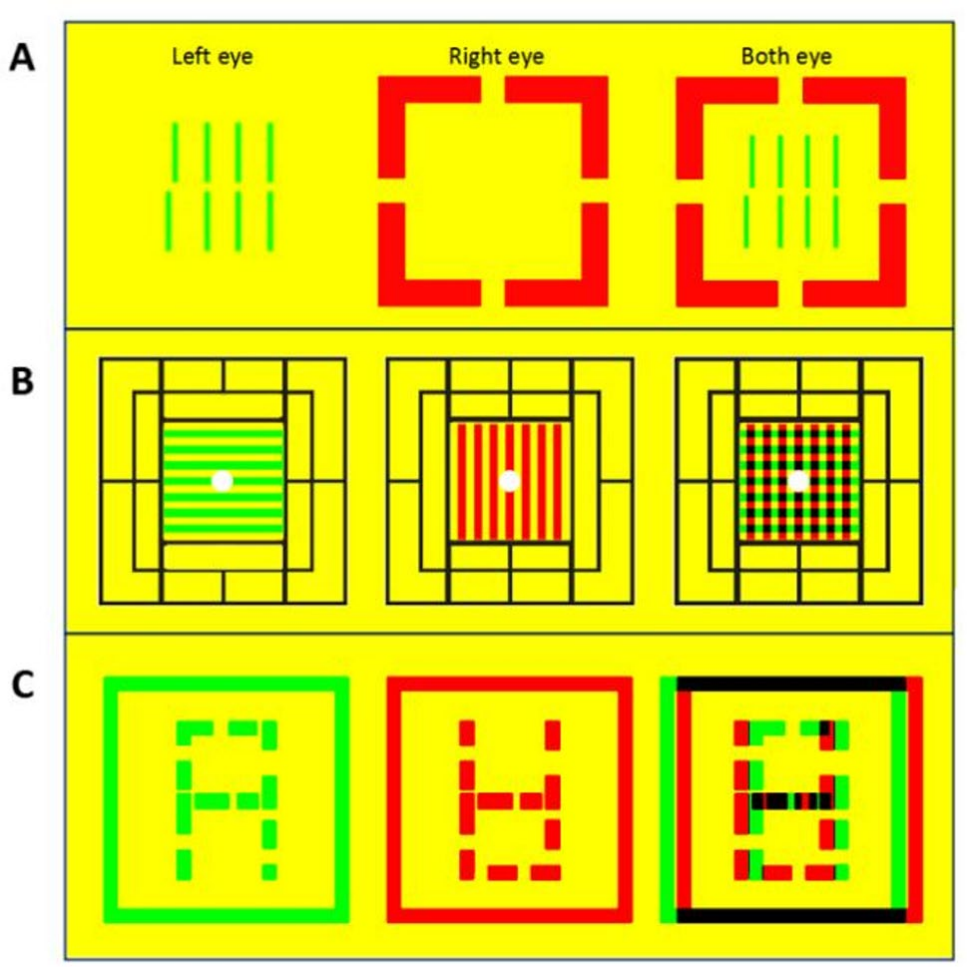
Competitive-state visual task testing methods. **A**: Competitive-state simultaneous perception test. The peripheral frame is visible to both eyes for fusion lock, while the central horizontal line is visible only to the left eye and the vertical line only to the right eye. **B**-**C**: Competitive-state fusion and stereopsis tests. The images presented to the left and right eyes exhibit complementary contour differences, with dynamic variations in binocular horizontal disparity

(1) Rivalry-Simultaneous Vision Test: The stimulus image consists of a surrounding frame (4.30°) and horizontal and vertical lines in the center (2.51°). The surrounding frame is visible to both eyes for fusion locking, with the horizontal line visible only to the left eye and the vertical line only to the right eye (Fig. 1B). If the participant can see both the horizontal and vertical lines simultaneously or alternately, they pass this test.

(2) Rivalry-Fusion Vision Test: The left eye sees an image consisting of a frame (4.30°) and an ‘A’ shaped visual marker (3.22°), while the right eye sees the same frame with a complementary outline of an inverted ‘A’ visual marker (Fig. 1C). If the participant can see a number 8 formed by the fusion of the two images, they pass this test.

(3) Rivalry-Stereoscopic Vision Test: The stimulus image, as in Fig. 1C, involves the frames in the images for the left and right eyes moving horizontally in alternating and non-alternating patterns (speed 0.14°/s, range of movement 0.36°, frequency 0.5 Hz), while the number 8 in the images moves horizontally in the opposite pattern to the frame, creating a perceptual sense of depth for the number 8 and the frame (total disparity range 0-0.72°). If the participant can judge the relative depth of the number 8 in relation to the surrounding frame, they pass this test [6].

## Statistical methods

Data analysis was performed using SPSS 23.0 statistical software. Categorical data (e.g., defect rates of various visual functions) are expressed as “n (%)”. The chi-square test (χ²) was used to analyze the defect rates in each visual function test between myopic and non-myopic students, as well as the correlations of age and gender with visual function defects. The Bonferroni correction was applied for post-hoc pairwise comparisons. No further adjustment for multiple comparisons across different visual function tests was performed, as these were pre-specified, distinct outcomes. Continuous data (e.g., SE values, vernier acuity disparity) are expressed as “mean ± standard deviation (x̄ ± s)”. Intergroup comparisons were conducted using one-way analysis of variance (ANOVA).

Correlation analysis: The Pearson correlation coefficient was used to evaluate the strength of association between myopia severity (SE value) and fusion function defects. Data normality was confirmed using the Shapiro-Wilk test prior to analysis. Significance level: A value of *P* < 0.05 was considered statistically significant. B-C: Competitive-state fusion and stereopsis tests. The images presented to the left and right eyes exhibit complementary contour differences, with dynamic variations in binocular horizontal disparity.

## Research results

## Analysis of the association between fusion function and myopia

### Differences in fusion function between myopic and non-myopic students

The fusion function defect rate in myopic students was 35.8% (256/715), significantly higher than the 26.6% (190/715) in non-myopic students, with a statistically significant difference (χ² = 14.194, *P* < 0.001). Further calculation of the odds ratio revealed that myopic students had a 1.54 times higher risk of fusion function defects compared to non-myopic students (OR = 1.54, 95% CI: 1.22–1.94), indicating a significant association between myopia status and fusion function defects (Table 1).

**Table 1:** Analysis of the association between fusion function defects and myopia in students

Fusion Function Status	Myopia (*n*)	Non-myopia (*n*)	Total (*n*)	χ² value	*P* value	OR(95%CI)
Normal	459	525	984	14.194	< 0.001	1.00(reference)
Abnormal	256	190	446			1.54(1.22 ~ 1.94)
Total	715	715	1430			

Note: The OR values used the “non-myopia + normal fusion function” group as the reference. *P* < 0.001 indicates that the difference is statistically highly significant. ^*^The ‘Non-myopia’ group here includes all students not meeting the clinical myopia criterion (SE > -0.50 D), encompassing those with low hyperopic reserve. For precise stratification by refractive error in subsequent tables (Tables 2, 4 and 5), students at the diagnostic threshold were excluded ^*^

### Association between different myopia severity levels and fusion function

The distribution of different myopia severity levels (non-myopia group, low myopia group, moderate myopia group, high myopia group) in the normal and abnormal fusion function groups was statistically significant (χ² = 13.742, *P* = 0.003). The distribution trends showed: In the normal fusion function group, low myopia accounted for the highest proportion (394/984 = 40%), followed by the non-myopia group (495/984 = 50.3%). In the abnormal fusion function group, moderate myopia accounted for the highest proportion (221/446 = 49.6%), followed by the low myopia group (178/446 = 39.9%). Correlation analysis revealed a weak positive linear association between increasing myopia severity and fusion defects, with a Pearson correlation coefficient of 0.087 (*P* < 0.01). The coefficient of determination (r² = 0.0076) suggests that myopia severity explains only about 0.76% of the variation in fusion defects. While statistically significant, this correlation is very weak in magnitude, implying that myopia severity alone is not a clinically meaningful predictor of fusion function status in this population. (Tables 2 and 3).

**Table 2:** Analysis of the association between different myopia severity levels and fusion function defects

Fusion Function Status	Non-Myopia Group (*n*)	Low Myopia Group (*n*)	Moderate Myopia Group (*n*)	High Myopia Group (*n*)	Total (*n*)	χ² Value	*P* Value	OR (Moderate vs. Non-Myopia, 95% CI)
Abnormal	178	178	221	3	446	-	-	2.12(1.63 ~ 2.76)
Normal	495	394	89	6	984	13.742	0.003	1.00(Reference)
Total	673	572	310	9	1430	-	-	-

Note: OR values used the “Non-Myopia + Normal Fusion Function” group as the reference. The moderate myopia group showed a significantly higher risk of fusion defects compared to the non-myopia group (*P* = 0.003). The high myopia group was not included in OR calculations due to small sample size (*n* = 9)

**Table 3:** Correlation analysis between myopia severity and fusion function defects

Indicator	Myopia Severity (SE Value)	Fusion Function Defect (Value: 0 = Normal, 1 = Abnormal)
Myopia Severity	1.000	-
Fusion Function Defect	0.087**	1.000

Note: indicates *P* < 0.01. The Pearson correlation coefficient *r* = 0.087 suggests a weak positive linear relationship between the two variables (r² = 0.0076). Although statistically significant, the very low r² value indicates that myopia severity accounts for less than 1% of the variability in fusion defects, highlighting its limited clinical utility as a sole indicator of fusion function impairment

## Analysis of the association between simultaneous perception, stereopsis, and myopia

### Association between stereopsis and myopia

The stereopsis defect rate was 44.1% (315/715) in myopic students and 46.6% (333/715) in non-myopic students, showing no statistically significant difference (χ² = 0.646, *P* = 0.886). Furthermore, the distribution of myopia severity levels did not differ significantly between normal and abnormal stereopsis groups (all *P* > 0.05). This indicates no significant association between myopia status or severity and stereopsis defects in this population (Table 4).

**Table 4:** Analysis of the association between different myopia severity levels and stereopsis function defects

Stereopsis Function Status	Non-Myopia Group (*n*)	Low Myopia Group (*n*)	Moderate Myopia Group (*n*)	High Myopia Group (*n*)	Total (*n*)	χ² Value	*P* Value
Abnormal	301	283	59	5	648	-	-
Normal	372	332	74	4	782	0.646	0.886
Total	673	572	310	9	1430	-	-

Note: *P* = 0.886 > 0.05, indicating no statistically significant association between different myopia severity levels and stereopsis function defects

**Table 5:** Analysis of the association between different myopia severity levels and simultaneous perception function defects

Simultaneous Perception Status	Non-Myopia Group (*n*)	Low Myopia Group (*n*)	Moderate Myopia Group (*n*)	High Myopia Group (*n*)	Total (*n*)	χ² Value	*P* Value
Abnormal	269	262	42	5	578	-	-
Normal	404	353	91	4	852	6.445	0.092
Total	673	572	310	9	1430	-	-

Note: *P* = 0.092 > 0.05, indicating no statistically significant association between different myopia severity levels and simultaneous perception function defects

### Association between simultaneous perception and myopia

The simultaneous perception defect rate was 41.5% (297/715) in myopic students and 39.3% (281/715) in non-myopic students, with no statistically significant difference between the two groups (χ² = 6.445, *P* = 0.092). Similarly, no significant differences were found in the distribution of myopia severity levels between groups with normal and abnormal simultaneous perception. These results indicate no significant association between myopia and simultaneous perception defects (Table 5).

## Competitive binocular visual function: age and gender differences

### Age impact on fusion function

Participants were divided into a lower-grade group (7–9 years, *n* = 721) and a higher-grade group (10–12 years, *n* = 709). The results showed: The fusion defect rate in the lower-grade group was 50.6% (365/721), significantly higher than the 11.4% (81/709) in the higher-grade group. The chi-square test indicated an extremely statistically significant difference between the two groups (χ² = 255.947, *P* < 0.001). Odds ratio analysis revealed that the lower-grade group had an 8.05 times higher risk of fusion defects compared to the higher-grade group (OR = 8.05, 95% CI: 6.18–10.49), suggesting that younger age is associated with a higher risk of fusion function defects Table 6). A further subgroup analysis stratified by myopia status confirmed that this age-related trend was consistent across both myopic and non-myopic students.

**Table 6:** Analysis of the association between different age groups and fusion function defects

Fusion Function Status	7–9 Years (*n*)	10–12 Years (*n*)	Total (*n*)	χ² Value	*P* Value	OR(95%CI)
Abnormal	365	81	446	-	-	8.05(6.18 ~ 10.49)
Normal	356	628	984	255.947	< 0.001	1.00(Reference)
Total	721	709	1430	-	-	-

Note: OR values used the “10–12 years + normal fusion function” group as the reference. *P* < 0.001 indicates that the difference is statistically highly significant

### Gender impact on fusion function

The fusion defect rate was significantly higher in males (46.9%, 332/708) than in females (15.8%, 114/722) (χ² = 161.134, *P* < 0.001). Males had a 4.63 times higher risk of fusion defects compared to females (OR = 4.63, 95% CI: 3.64–5.89), identifying gender as a significant factor associated with fusion function outcomes (Table 7).

**Table 7:** Analysis of the association between different genders and fusion function defects

Fusion Function Status	Male (*n*)	Female (*n*)	Total (*n*)	χ² Value	*P* Value	OR(95%CI)
Abnormal	332	114	446	-	-	4.63(3.64 ~ 5.89)
Normal	376	608	984	161.134	< 0.001	1.00(Reference)
Total	708	722	1430	-	-	-

Note: OR values used the “female + normal fusion function” group as the reference. *P* < 0.001 indicates that the difference is statistically highly significant

## Discussion

This cross-sectional study systematically analyzed the association between binocular visual function and myopia in 1,430 elementary school students using an innovative dynamic screening protocol. The key finding is a significant and specific association between myopia and defects in fusion function, independent of simultaneous perception or stereopsis. This supports the perspective that myopia involves more than refractive error, extending to aspects of visual information processing. It is crucial to emphasize that this study design can only establish an association, not causality. The pathophysiological mechanisms and clinical implications warrant further investigation.

This study found that after refractive correction, myopic children exhibited a significantly higher rate of fusion function defects compared to non-myopic children, with the severity of defects positively correlated with the degree of myopia. Children with high myopia showed a further increase in defect rates. Further stratified analysis revealed that the moderate myopia group accounted for the highest proportion (49.55%) among those with fusion defects, and a weak positive linear correlation was observed between myopia severity and fusion defects (*r* = 0.087, *P* < 0.01). This suggests that the association between myopia severity and fusion defects may be modulated by multiple factors. Fusion function abnormalities are not solely caused by refractive status but may represent an external manifestation of insufficient central visual system integration capability. Fusion vision relies on precise matching of binocular retinal information and coordinated processing of neural signals. During myopia progression, axial elongation-induced retinal image displacement and accommodation-convergence imbalance may further increase the burden on central visual integration, forming a bidirectional cycle of “myopia progression - fusion defects.” On the other hand, prolonged near-work activities (such as reading and electronic screen use) in children continuously activate the accommodation-convergence reflex. If the accommodation speed and vergence amplitude between the two eyes are mismatched, fusion function may remain in a compensatory state for extended periods, eventually leading to defects. This result also explains why some non-myopic students still exhibit fusion defects (26.57%), indicating that fusion function screening should not be limited to myopic populations but should cover children of all ages. However, this cross-sectional evidence only confirms a correlation. Early intervention for fusion defects may serve as a potential target for delaying myopia onset, especially for groups with a family history of myopia or high near-work visual loads [7–10].

From an age perspective, younger children (7–9 years old) showed a significantly higher risk of fusion function defects compared to older children (10–12 years old), suggesting that early childhood may be a critical period for visual function intervention. Ages 7–9 represent a transitional phase where children’s refractive status shifts from hyperopic reserve to emmetropia, while academic demands increase near-work time, abruptly elevating the need for accommodation-convergence coordination. If the central visual system has not yet established a stable vergence control mechanism during this period, insufficient compensation of fusion function may easily occur, manifesting as high defect rates. In contrast, children aged 10–12 have largely mature fusion function, and with age, accumulated visual experience and improved neural regulation efficiency lead to a significant decline in defect rates. This suggests that clinical practice should prioritize children aged 7–9 for fusion function screening. Early visual training to promote visual function maturation may indirectly reduce the risk of myopia onset [11–14].

Gender difference analysis indicated that females outperformed males in fusion function, consistent with previous research on gender differences in visual function. This may be related to physiological differences in visual cortex development: females exhibit higher neuronal density in the visual cortex, faster integration of binocular information, and greater stability in accommodation-convergence reflexes. Female children demonstrate stronger attentional control and perceptual integration capabilities in visual tasks, while males are more susceptible to multitasking interference. Additionally, although male children may spend more time outdoors, they often demonstrate poorer postural discipline during near-work activities (e.g., head tilting, monocular viewing), which may long-term lead to binocular vergence imbalance and increased fusion function burden [15–18].

In contrast to fusion function, no significant differences were observed in simultaneous perception and stereopsis between myopic and non-myopic children. Binocular visual function develops in the sequence of “simultaneous perception, fusion, stereopsis.” Simultaneous perception involves basic judgment of the “presence or absence” of binocular information and matures early (typically before age 6) and are relatively stable in our 7-12-year-old cohort. Stereopsis represents advanced depth perception integration. Although stereopsis relies on fusion function, once established, it remains relatively stable and is less directly affected by acquired refractive changes. The participants in this study were aged 7–12, having already passed the critical developmental windows for simultaneous perception and stereopsis. Their functional status is more likely influenced by early visual conditions (e.g., strabismus, amblyopia) rather than current myopia status. Moreover, this study employed “competitive tasks” to simulate complex visual environments. Compared to traditional static tests, visual interference in complex environments (such as the binocular competitive stimuli used here) amplifies minor anomalies in vergence function. In contrast, simultaneous perception tests only require recognition of the “presence or absence” of binocular stimuli, while stereopsis tests rely on depth perception formed by fixed disparities, both being less dependent on dynamic vergence. Thus, they showed no association with myopia [19–22].

It is important to note that due to the cross-sectional nature of this study, the observed associations between fusion function deficits, myopia, age, and gender cannot establish causality or temporal sequence. The relationships reported here are associative, and causal inferences would require longitudinal or interventional study designs. This study has several limitations: (1) It can only reveal an association between fusion function defects and myopia, not establish causality. Longitudinal cohorts are needed to clarify the temporal sequence. (2) Refractive measurement bias: Although non-cycloplegic refraction was used in this study to reflect children’s daily refractive status, it may overestimate myopia severity (particularly in younger children with strong accommodative ability), potentially introducing bias in defining the “non-myopia group.” Future studies should incorporate cycloplegic refraction to enable more precise analysis of the relationship between refractive status and fusion function. (3) The study included students from only one elementary school in Wuxi, lacking regional diversity. The generalizability of results to regions with different economic levels and educational models across China requires validation. (4) Inadequate control of confounding factors: Factors such as eye habits, outdoor activity time, and family history of myopia were not fully excluded, potentially affecting the accuracy of conclusions. (5) Further validation of the dynamic screening protocol is needed: Although the competitive-state visual tasks used in this study were designed based on sound visual principles and showed preliminary test-retest reliability, their comprehensive criterion validity against classical clinical examinations (e.g., Worth four-dot test, Titmus stereo test) has not been systematically established in large samples. Future studies should directly compare this dynamic screening protocol with traditional methods to further establish its clinical validity and diagnostic thresholds.

In conclusion, this cross-sectional study identifies a significant association between competitive binocular fusion function deficits and myopia in school-aged children, with stronger associations observed in younger children and males. These associative findings highlight fusion function as a potential area of interest in myopia research. At a clinical level, they suggest that assessment of fusion function, particularly in younger children, might provide additional information for identifying children who could benefit from comprehensive visual assessments. Regarding practical implications, these correlative findings suggest that incorporating fusion function screening into existing school vision health programs may help provide a more comprehensive assessment of a child’s visual health risk. 

## Supplementary Information

Below is the link to the electronic supplementary material.

Supplementary Material 1

## Data Availability

The datasets generated and analyzed during the current study are available in its supplementary information files.

## Abbreviations

OR: Odds Ratio
CI: Confidence Interval
AC/A: Accommodative Convergence / Accommodation Ratio
CV: Coefficient of Variation
SE: Spherical Equivalent
IMI: International Myopia Institute
BCVA: Best Corrected Visual Acuity
ANOVA: Analysis of Variance

## References

[1] Flitcroft DI, He M, Jonas JB, Jong M, Naidoo K, Ohno-Matsui K, et al. IMI – Defining and classifying myopia: A proposed set of standards for clinical and epidemiologic studies. Invest Ophthalmol Vis Sci. 2019;60(3):M20–30. 10.1167/iovs.18-25957. 30817826 10.1167/iovs.18-25957 PMC6735818

[2] Sankaridurg P, Berntsen DA, Bullimore MA, Cho P, Flitcroft I, Gawne TJ, et al. IMI 2023 digest. Invest Ophthalmol Vis Sci. 2023;64(13):23. 10.1167/iovs.64.13.23. 10.1167/iovs.64.6.7 PMC10155872 37126356

[3] Zaabaar E, Asiamah R, Kyei S, Ankamah S. Myopia control strategies: A systematic review and meta-meta‐analysis. Ophthalmic Physiol Opt. 2024;44(4):749–65. 10.1111/opo.13312. 10.1111/opo.13417 39530399

[4] Lawrenson JG, Shah R, Huntjens B, Downie LE, Virgili G, Dhakal R, et al. Interventions for myopia control in children: a living systematic review and network meta-analysis. Cochrane Database Syst Rev. 2023;2023(2):CD014758. 10.1002/14651858.CD014758.pub2. 10.1002/14651858.CD014758.pub2 PMC9933422 36809645

[5] Mutti DO, Mitchell GL, Hayes JR, Jones LA, Moeschberger ML, Cotter SA, et al. Accommodative lag before and after the onset of myopia. Invest Ophthalmol Vis Sci. 2006;47(3):837–46. 10.1167/iovs.05-0888. 16505015 10.1167/iovs.05-0888

[6] Chen YJ, Zhang XH, et al. An investigation into the correlation between visual performance in simulated complex environments and academic attainment among primary school students. Sci Rep. 2024;14:5879. 10.1038/s41598-024-56411-9. 38467774 10.1038/s41598-024-56548-7 PMC10928190

[7] Vedamurthy I, Nahum M, Huang SJ, Zheng F, Bayliss J, Bavelier D, et al. A dichoptic custom-made action video game as a treatment for adult amblyopia. Vis Res. 2015;114:173–83. 10.1016/j.visres.2015.04.008. 25917239 10.1016/j.visres.2015.04.008 PMC4549206

[8] Romero MC, Van Dromme IC, Janssen P, et al. The role of binocular disparity in stereoscopic images of objects. PLoS ONE. 2013;8(2):e55340. 10.1371/journal.pone.0055340. 23408970 10.1371/journal.pone.0055340 PMC3567065

[9] Cronström A, Creaby MW, Nae J, Ageberg E. Gender differences in visual task performance: A Meta-Analysis. Front Psychol. 2020;11:552. 10.3389/fpsyg.2020.00552. 32373001 10.3389/fpsyg.2020.00552 PMC7187750

[10] Mutti DO, Sinnott LT, Mitchell GL, Jones-Jordan LA, Moeschberger ML, Cotter SA, et al. Relative peripheral refractive error and the risk of onset and progression of myopia in children. Invest Ophthalmol Vis Sci. 2011;52(1):199–205. 10.1167/iovs.10-5459. 20739476 10.1167/iovs.09-4826 PMC3053275

[11] Simons K, Elhatton K. Stereoacuity and functional vision. Am J Ophthalmol. 2005;139(3):550–3. 10.1016/j.ajo.2004.09.070.15767075

[12] Shankar S, Evans MA, Bobier WR. Hyperopia and emergent literacy of young children: pilot study. Optom Vis Sci. 2007;84(11):1031–8. 10.1097/OPX.0b013e318157a67a. 18043422 10.1097/OPX.0b013e318157a67a

[13] Mavi S, Chan VF, Virgili G, Biagini I, Congdon N, Piyasena P, et al. The impact of hyperopia on academic performance among children: A systematic review. Asia Pac J Ophthalmol (Phila). 2022;11(1):36–51. 10.1097/APO.0000000000000488. 35066525 10.1097/APO.0000000000000492

[14] Orlansky G, Hopkins KB, Mitchell GL, Huang K, Frazier M, Heyman C, et al. Reliability of the developmental eye movement test. Optom Vis Sci. 2011;88(12):1507–19. 10.1097/OPX.0b013e31822b1a70. 21964661 10.1097/OPX.0b013e318230f03a

[15] Vinuela-Navarro V, Erichsen JT, Williams C, Woodhouse JM. Saccades and fixations in children with delayed reading skills. Ophthalmic Physiol Opt. 2017;37(4):531–41. 10.1111/opo.12388. 28656674 10.1111/opo.12392

[16] Parker AJ. Binocular depth perception and the cerebral cortex. Nat Rev Neurosci. 2007;8(5):379–91. 10.1038/nrn2131. 17453018 10.1038/nrn2131

[17] van Dam LC, van Ee R. The role of saccades in exerting voluntary control in perceptual and binocular rivalry. Vis Res. 2006;46(6–7):787–99. 10.1016/j.visres.2005.10.011. 16309727 10.1016/j.visres.2005.10.011

[18] Brych M, Murali S, Händel B. The role of Blinks, microsaccades and their retinal consequences in bistable motion perception. Front Psychol. 2021;12:647256. 10.3389/fpsyg.2021.647256. 33897552 10.3389/fpsyg.2021.647256 PMC8061730

[19] Cox MA, Dougherty K, Westerberg JA, Schall MS, Maier A. Temporal dynamics of binocular integration in primary visual cortex. J Vis. 2019;19(12):13. 10.1167/19.12.13. 31622471 10.1167/19.12.13 PMC6797477

[20] Mitchell BA, Dougherty K, Westerberg JA, Carlson BM, Daumail L, Maier A, et al. Stimulating both eyes with matching stimuli enhances V1 responses. iScience. 2022;25(5):104182. 10.1016/j.isci.2022.104182. 35494250 10.1016/j.isci.2022.104182 PMC9038564

[21] Andrews TJ, Holmes D. Stereoscopic depth perception during binocular rivalry. Front Hum Neurosci. 2011;5:99. 10.3389/fnhum.2011.00099. 21960966 10.3389/fnhum.2011.00099 PMC3177177

[22] Cai T, Zhu H, Xu J, Wu S, Li X, He S. Human cortical neural correlates of visual fatigue during binocular depth perception: an fNIRS study. PLoS ONE. 2017;12(2):e0172426. 10.1371/journal.pone.0172426. 28207899 10.1371/journal.pone.0172426 PMC5312944

